# Clinical characteristics of bloodstream infections in adult patients with solid tumours and a nomogram for mortality prediction: a 5-year case-controlled retrospective study in a tertiary-level hospital

**DOI:** 10.3389/fcimb.2023.1228401

**Published:** 2023-08-08

**Authors:** Lijuan Xue, Ying Zhu, Mingxi Zong, Panpan Jiao, Jianguo Fu, Xian-Ming Liang, Juan Zhan

**Affiliations:** ^1^ Department of Oncology Medicine, Zhongshan Hospital of Xiamen University, School of Medicine, Xiamen University, Xiamen, China; ^2^ School of Medicine, Xiamen University, Xiamen, China; ^3^ School of Pharmacy, Xiamen University, Xiamen, China; ^4^ Department of Nosocomial Infection and Preventive Health Care, Zhongshan Hospital of Xiamen University, School of Medicine, Xiamen University, Xiamen, China; ^5^ Center of Clinical Laboratory, Zhongshan Hospital of Xiamen University, School of Medicine, Xiamen University, Xiamen, China; ^6^ Institute of Infectious Disease, School of Medicine, Xiamen University, Xiamen, China

**Keywords:** bloodstream infections, mortality, nomogram, risk factors, solid tumors

## Abstract

**Background:**

Bloodstream infections (BSIs) are one of the leading causes of death in cancer patients. Nevertheless, the risk factors of BSIs in solid tumors have rarely been ascertained adequately.

**Methods:**

We conducted a single-center case-controlled retrospective study from 2017 to 2021 among adults with solid tumors in a tertiary-level hospital. The BSIs and control group were matched by the propensity score matching method. We found independent risk factors of occurrence and death of BSIs using univariate and multivariate regression analysis. Additionally, a nomogram was constructed to predict the risk of mortality in BSIs.

**Results:**

Of 602 patients with solid tumors in the study period, 186 had BSIs and 416 had non-BSIs. The incidence of BSIs was 2.0/1,000 admissions (206/102,704), and the 30-day mortality rate was 18.8% (35/186). Compared to the control group, the BSIs had longer hospital stays (24.5 days vs. 20.0 days), and higher frequency complicating with organ failure (10.5% vs. 2.4%), nephropathy (19.6% vs. 3.8%), comorbidities≥3 (35.5% vs. 20.0%), and liver-biliary-pancreatic infections (15.6% vs. 5.3%) (all P<0.001). Among the 186 patients with BSIs, 35 died within 30 days after BSIs. Gram-negative bacteria were the most frequent microorganisms (124/192, 64.6%). Liver cancer, organ failure, a high level of lactate dehydrogenase and septic shock were the independent hazardous factors for death of BSIs. What’s more, a nomogram was constructed to predict the 30-day survival rate of BSIs, which was proved to have good accuracy (AUC: 0.854; 95% confidence interval: 0.785~0923) and consistency.

**Conclusion:**

Being aware of the risk factors of BSIs redounds to take preventive measures to reduce the incidence and death of BSIs.

## 1 Introduction

Globally, the incidence of malignant tumors is about 100 per 100,000 people per year (Okongo et al., 2019; Lin et al., 2021), which is increasing every year (Ryzhov et al., 2020) and may become the main cause of death in many regions of the world (Ben-Batalla et al., 2019). Bloodstream infections (BSIs) are one of the most important causes of death in cancer patients (Sierra et al., 2020). BSIs refer to pathogenic microorganisms invading the human body, entering blood circulation and spreading throughout the body resulting in bacteremia, sepsis, septic shock, and even death. The incidence of BSIs in malignant tumors is about 5.5 per 1,000 admissions (Valentine et al., 2020) and the mortality rate is significantly higher than that of patients without BSIs (10.4% vs. 1.8%) (Kitaya et al., 2022). Moreover, the 30-day mortality rate of *Escherichia coli* BSI in solid tumors is even as high as 35% (Zhang et al., 2019), which is quite astonishing.

Several investigations had revealed that the incidence of BSIs in hematologic malignancies is higher than that in solid tumors (Sierra et al., 2020; Royo-Cebrecos et al., 2022). Currently, a large number of studies associated with BSIs have concentrated on hematologic malignancies. Although the prevalence of BSIs in solid tumors is lower, the overall incidence of solid tumors far exceeds that of hematologic malignancies (Al-Otaibi et al., 2016). BSIs occur in about 5.5-16.4% of patients with solid tumors (Gudiol et al., 2016). But studies focused on BSIs with solid tumors are limited. Therefore, it is necessary to further investigate the clinical characteristics of BSIs in patients with solid tumors and explore the risk factors for the incidence and mortality of BSIs. Thus, we designed the study to comprehensively unravel the epidemiology, clinical characteristics, and laboratory manifestation of patients with BSIs in solid tumors to get an overview of the current condition of BSIs.

## 2 Materials and methods

### 2.1 Population selection and setting

The study was a single-center case-controlled retrospective study including 186 patients with BSIs in solid tumors who were treated in Zhongshan Hospital affiliated to Xiamen University from January 2017 to December 2021. This is a tertiary-level hospital integrating medical treatment, teaching, prevention and health care. It admits more than 60,000 inpatients every year, including over 10,000 patients with malignant tumors.

**Figure 1:**
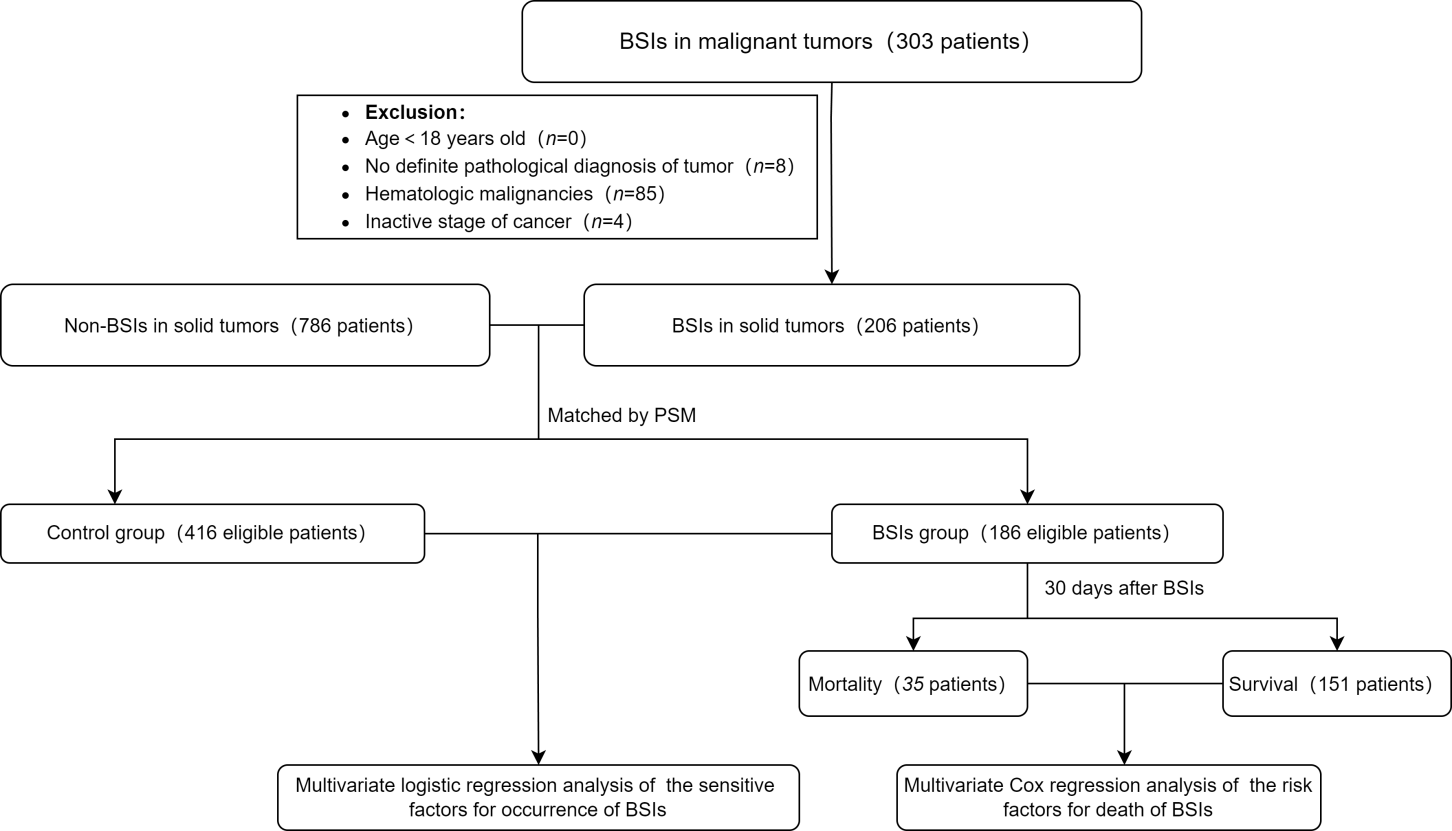
Flowchart of the study. The controls were selected from the patients without BSIs. 206 cases in the BSIs group were matched with 786 cases in the control at a ratio of 1:3. Based on the matching conditions which involved age, sex and tumor types, 20 cases in the BSIs group were removed automatically and some were matched at a ratio of 1:2 because of using nearest neighbor matching by a caliper of 0.1 standard deviations. *BSIs*, bloodstream infections; *PSM*, Propensity Score Matching method.

This study included only solid tumors with BSIs. Eligible participants included all patients ≥ 18 years old who had suffered at least one episode of BSIs. Their cancers were in the active stage. Based on the definition of BSIs by the Centers for Disease Control and Prevention (Garner et al., 1988), the inclusion criteria of the BSIs group were as follows: (i) The patient’s body temperature was more than 38.0 °C or below 36.0 °C, or patient was accompanied by chills; (ii) Blood culture was positive (but normal skin flora, such as *diphtheria*, *Bacillus* spp., *Propionibacterium* spp., and *micrococci* need to two blood cultures at different times); (iii) At least one positive blood culture obtained at the same hospitalization and the first infection was recorded; (iv) The pathological diagnosis was identified as a malignant tumor. The above four criteria need to be met at the same time in the study. Finally, there were 186 patients of BSIs and 416 of non-BSIs enrolled in this study. **Figure 1** showed the procedure of the study.

### 2.2 Definitions

According to CDC definitions for BSIs (Garner et al., 1988), laboratory-confirmed BSIs were defined as primary BSIs if the pathogen isolated from the blood culture was not associated with other sites of infection, otherwise they were secondary BSIs. According to the definition of catheter-related BSI (CRBSI) in the Infectious Diseases Society of America (IDSA) (Mermel et al., 2009), we defined CRBSI as follows: (i)Semi-quantitative or quantitative culture of catheter colonies was positive; (ii)Semi-quantitative culture of strains was positive, or the count of cultured strains in blood samples taken from the central vein and peripheral vein at a ratio of 5:1 increased obviously; (iii)Both blood cultures from central vein and peripheral vein at different times were positive. Pathogen which showed insensitivity to 3 or more types of antibacterial drugs was defined as multi-drug resistance (MDR) (Kadri et al., 2018). Chemotherapy and surgery history were all traced back to one month before BSIs. The history of intensive care unit (ICU) admission meant transferring to ICU due to BSIs during the same hospitalization. Neutrophil-to-lymphocyte (NLR) referred to the ratio of neutrophil count to lymphocyte count. Inappropriate initial antibiotic therapy (IIAT) was defined as the antibiotics used within 72 hours after suspecting BSIs but proved to be inactive against the pathogen *in vitro* culture and susceptibility testing (Tang et al., 2020).

### 2.3 Data collection

The information of all participants was collected from the electronic medical record system and the doctors’ order system. The demographic and clinical features of the patients included age, sex, body mass index (BMI), length of stay in hospital, laboratory examination, characteristics of infections, comorbidities and use of antibiotics, etc. The susceptibility results of antibiotics were collected from the laboratory information system. The incidence was calculated from annual admission data, which was displayed as the number of BSIs episodes per 1000 hospitalized patients.

### 2.4 Statistical analysis

The matching of the BSIs and control group was done by the Propensity Score Matching (PSM) plugin. All analyses were carried out by SPSS26.0 (IBM SPSS Statistics, IBM Corp., Armonk, NY, US). The continuous variables which were normally distributed were described as mean ± standard deviation, and differences between the BSIs and control group were tested by T-test. While those that did not follow normal distribution were represented by median (25-75% quartile ranges), using the Manne Whitney U test. The classification variables were expressed as frequency and percentage, and the differences between the two groups were compared by the chi-square test or Fisher exact test. The Cox regression model was used to determine the hazard factors for death of BSIs in solid tumors, and constructed by R. Statistics 4.2.3 software. The statistical difference was defined as *P*<0.05 (double tails).

## 3 Results

### 3.1 Clinical characteristics of patients

From 2017 to 2021, there were 102,704 admissions in total with patients who visited Zhongshan Hospital affiliated to Xiamen University for solid tumors. A total of 303 patients with BSIs in malignant tumors, of which 97 were excluded for the following reasons: (i) There was no definite pathological diagnosis of tumor (n=8); (ii) The patient was diagnosed as hematologic malignancies (n=85); (iii) Cancer had been cured clinically(n=4). Finally, the number of patients included in the study was 206. The BSIs and control cohorts were matched in a ratio of 1:3 by PSM. The matching conditions involved age, sex and tumor types. The demographic and clinical features of BSIs and control cohorts were displayed in **Table 1**. The median age was 62 and 61 years old, while males accounted for 72.0% and 72.6%, respectively. There were no significant differences in age, sex and tumor types between the two cohorts.

**Table 1:** Demographic and clinical characteristics of patients in the BSIs and control groups.

Variables	BSIs N=186, n (%)	Control N=416, n (%)	*P* Value
**Age (years)**	61.8 ± 11.2	61.2 ± 10.0	0.51
**Sex (male)**	134(72.0)	302(72.6)	0.888
**BMI(kg/m^2^)**	20.59 (18.41~23.27)	21.30(19.14~23.57)	0.069
Types of tumors			
Head/neck/esophageal cancer	15(8.1)	32(7.7)	0.875
Lung cancer	8(4.3)	21(5.0)	0.692
Breast cancer	4(2.2)	14(3.4)	0.419
Gastric cancer	31(16.7)	73(17.5)	0.792
Liver cancer	37(19.9)	99(23.8)	0.290
Ampullary/gallbladder/biliary tract/pancreatic cancer	28(15.1)	40(9.6)	0.051
Colon cancer	28(15.1)	67(16.1)	0.744
Rectal cancer	23(12.4)	44(10.6)	0.519
Genitourinary cancer ^a^	18(9.7)	32(7.7)	0.415
**Stage III/IV**	136(73.1)	280 (67.3)	0.154
**Multiple primary carcinomas**	5(2.7)	15(3.6)	0.562
Comorbidities			
Organ failure ^b^	16(10.5)	7(2.4)	<0.001^*^
Cardiovascular diseases ^c^	57(37.3)	119(41.3)	0.611
Pulmonary diseases ^d^	22(14.4)	54(18.8)	0.694
Digestive diseases ^e^	53(34.6)	90(31.3)	0.068
Diabetes mellitus	32(20.9)	64(22.2)	0.573
Nephropathy ^f^	30(19.6)	11(3.8)	<0.001^*^
Rheumatism	9(5.9)	12(4.2)	0.227
Hypoproteinemia	93(60.8)	99(34.4)	0.164
Anemia	37(24.2)	76(26.4)	0.637
Myelosuppression	6(3.9)	17(5.9)	0.611
Others ^g^	8(4.3)	7(1.7)	0.057
**Comorbidities ≥3**	66(35.5)	83(20.0)	<0.001^*^
Site of infection			
Upper respiratory tract	3(1.6)	8(1.9)	0.793
Lung/thorax	47(25.3)	105(25.2)	0.994
Gastrointestinal tract	8(4.3)	17(4.1)	0.903
Peritoneal cavity	14(7.5)	12(2.9)	0.01^*^
Liver/biliary tract/pancreas	29(15.6)	22 (5.3)	<0.001^*^
Urinary tract	13(7.0)	15(3.6)	0.069
Incision	5(2.7)	8(1.9)	0.551
Others ^h^	4(2.2)	10(2.4)	0.849
**Chemotherapy history**	45(24.2)	116(27.9)	0.344
**Surgery history**	89(47.8)	152(36.5)	0.009^*^
**Indwelling catheter**	143(76.9)	265(64.5)	0.003^*^
Trachea cannula	14(7.5)	5(1.2)	<0.001^*^
Chest drains	11(5.9)	12(2.9)	0.073
Abdominal drains	31(16.7)	70(16.8)	0.961
Urine tube intubation	49(26.3)	67(16.1)	0.003^*^
Arteriovenous cannula	91(48.9)	193(46.4)	0.565
Dialysis	10(5.4)	5(1.2)	0.002^*^
Laboratory findings			
NLR	4.46 (2.56~7.78)	4.05(2.44~7.34)	0.103
Percentage of lymphocytes (%)	16.60(10.15~24.28)	17.50(10.80~25.70)	0.182
ALB(g/L)	34.46(29.89~39.52)	35.20(30.90~38.77)	0.194
LDH (U/L)	199.90(158.85~275.25)	199.20(164.30~271.40)	0.694
BUN (mmol/L)	5.14(3.98~6.71)	4.80(3.80~6.40)	0.153
Creatinine (μmol/L)	64.10(51.23~82.00)	65.20(51.60~79.40)	0.618
Potassium ions (mmol/L)	3.93(3.66~4.23)	4.01(3.66~4.30)	0.242
Sodium ions (mmol/L)	138.10(134.90~141.30)	138.90(135.50~141.20)	0.524
**Length of hospital stay**	24.5(14.8~37.0)	20.0(13.0~31.0)	<0.001^*^
**Died within 30 days**	35(18.8)	18(4.3)	<0.001^*^

a Genitourinary cancer included kidney cancer, bladder cancer, urothelial cancer, prostate cancer, ureteral cancer, cervical cancer, ovarian cancer, and endometrioid adenocarcinoma.

b Organ failure included respiratory failure, cardiac failure, liver failure, renal failure and multiple organ dysfunction syndrome.

c Cardiovascular diseases included hypertension, coronary heart disease, cardiac insufficiency, atrial fibrillation and pericardial effusion.

d Pulmonary diseases included chronic obstructive pulmonary disease, pulmonary hypertension, pleural effusion and interstitial lung disease.

e Digestive diseases included hepatic insufficiency, gastrointestinal bleeding, gastrointestinal obstruction, viral hepatitis, cirrhosis and ascites.

f Nephropathy included acute kidney injury and chronic kidney disease.

g Others included hyperthyroidism, hypothyroidism, disseminated intravascular coagulation and syphilis.

h Others included oral, breast, pelvic, crissum and skin.

^*^ P < 0.05.

Compared with the control group, the patients with BSIs were more likely to have 3 or more underlying diseases (35.5% vs. 20.0%), organ failure (10.5% vs. 2.4%) or renal diseases (19.6% vs. 3.8%), and die within 30 days (18.8% vs. 4.3%) (all *P*<0.001). There were significantly more patients in the BSIs group who suffered from hepato-biliary-pancreatic infections (15.6% vs. 5.3%, *P*<0.001) and peritoneal cavity infections (7.5% vs. 2.9%, *P*=0.01). During hospitalization, there were significantly more cases experiencing mechanical ventilation, urinary catheter indwelling, hemodialysis, and surgery in the BSIs group (all *P*<0.01). The length of hospitalization in the BSIs group was eminently longer than that in the control (24.5 days vs. 20.0 days, *P*<0.001). Baseline laboratory data before blood culture (including NLR, lymphocyte count, serum albumin [ALB], lactate dehydrogenase [LDH], blood urea nitrogen [BUN], creatinine, potassium ions, and sodium ions) were not statistically different between the two cohorts.

In the BSIs group, there were 124 cases of Gram-negative bacteria (GNB) BSIs, 53 cases of Gram-positive bacteria (GPB) BSIs and 15 cases of fungus BSIs, which involved 180 cases of monomicrobial infections and 6 cases of polymicrobial infections. The six polymicrobial infections included Extended-Spectrum-Beta-Lactamases (ESBL) -producing *Escherichia coli* plus *Candida tropicalis*, ESBL-producing *Escherichia coli* plus *Candida albicans*, ESBL-producing *Escherichia coli* plus *Enterococcus faecalis*, *Pseudomonas aeruginosa* plus *Enterococcus avium*, *Acinetobacter baumannii* plus *Staphylococcus haemolyticus*, ESBL-negative *Escherichia coli* plus *Aeromonas hydrophila*, respectively. **Table 2** showed the distribution of microorganism of 192 episodes and the important drug resistance bacteria that causing BSIs in solid tumors. In 192 episodes of BSIs, the GNB BSIs, GPB BSIs and fungus BSIs accounted for 64.6%, 27.6% and 7.8% respectively. The most predominant GNB were *Escherichia coli* (n=67) and *Klebsiella pneumoniae* (n=27), while the most common GPB were *Staphylococcus aureus* (n=16) and *Enterococcus* spp.(n=14). *Klebsiella pneumoniae* had the highest 30-day mortality (25.9%, 7/27), followed by *Enterococcus* spp. (21.4%, 3/14). In terms of drug susceptibility testing, multi-drug resistant Gram-negative bacteria (MDR-GNB) accounted for 46.0% (57/124). There was no significant difference in outcomes caused by multi-drug resistant bacteria in the mortality and survival group (40.0% vs. 32.2%, *P* = 0.381) (**Table 3**). In *Escherichia coli* isolates, the sensitivities to cefotaxime were 35.8%. The *Enterobacteriaceae* producing ESBL accounted for 53.8%. The isolates of *Klebsiella pneumoniae* were 100.0% sensitive to imipenem and meropenem. About 12.5% of *Staphylococcus aureus* were resistant to methicillin.

**Table 2:** The distribution of microorganisms and antibiotic resistance of the frequent episodes in the BSIs group.

Microorganisms	n/M, (%)
**Gram-negative**	124/192(64.6)
*Escherichia coli*	67/124(54.0)
* Klebsiella pneumoniae*	27/124 (21.8)
* Pseudomonas aeruginosa*	6/124 (4.8)
* Enterobacter* spp.	5/124 (4.0)
* Acinetobacter* spp.	5/124 (4.0)
* Bacteroides* spp.	5/124 (4.0)
* Clostridium* spp.	2/124 (1.6)
Others	7/124 (5.6)
**Gram-positive**	53/192(27.6)
* Staphylococcus aureus*	16/53(30.2)
CONS	6/53 (11.3)
* Enterococcus* spp.	14/53 (26.4)
* Streptococcus* spp.	11/53 (20.8)
Others	6/53 (11.3)
***Fungus***	15/192(7.8)
Antibiotic resistance	
Drug-resistance GNB	103/124(83.1)
Drug-resistance GPB	37/53(69.8)
MDR-GNB	57/124(46.0)
ESBL-producing *Enterobacteriaceae*	49/91(53.8)
Cefotaxime-resistant *Escherichia coli*	43/67(64.2)
Carbapenem-resistant *Klebsiella pneumoniae*	0/27(0.0)
Methicillin-resistant *Staphylococcus aureus*	2/16(12.5)
Ampicillin- resistant *Enterococcus* spp.	4/14(28.6)

MDR, Multidrug-resistant.

ESBL, Extended Spectrum Beta-Lactamases.

**Table 3:** Demographic and clinical characteristics of the mortality and survival cohorts in BSIs patients with solid tumors.

Variables	Mortality N=35, n (%)	Survival N=151, n (%)	*P* value
**Age (years)**	61.46 ± 11.20	61.83 ± 11.21	0.858
**Sex (male)**	23(65.7)	110(73.5)	0.354
**BMI (kg/m^2^)**	20.70 (18.21~22.68)	20.76 (18.43~23.46)	0.634
Types of tumors			
Head/neck/esophageal cancer	1(2.9)	14(9.3)	0.362
Lung cancer	2(5.7)	6(4.0)	0.627
Breast cancer	1(2.9)	3(2.0)	1.000
Gastric cancer	9(25.7)	22(14.6)	0.111
Liver cancer	12(34.3)	25(16.6)	0.018^*^
Ampullary/gallbladder/biliary tract/pancreatic cancer	2(5.7)	26(17.2)	0.086
Colon cancer	6(17.1)	22(14.6)	0.701
Rectal cancer	2(5.7)	21(13.9)	0.298
Genitourinary cancer ^a^	1(2.9)	17(11.3)	0.231
**Stage III/IV**	28(93.3)	108(76.1)	0.035^*^
**Multiple primary carcinomas**	1(2.6)	4(2.7)	0.981
**Chemotherapy history**	7(20.0)	38(25.2)	0.520
**Surgery history**	16(45.7)	73(48.3)	0.779
Underlying infection			
Any	13(37.1)	81(53.6)	0.079
Lung/thorax	15(42.9)	32(21.2)	0.008^*^
Gastrointestinal tract	4(11.4)	4(2.6)	0.065
Liver/biliary tract/pancreas	4(11.4)	25(16.6)	0.451
Peritoneal cavity	6(17.1)	8(5.3)	0.042^*^
Urinary tract	3(8.6)	10(6.6)	0.968
Comorbidities			
Any	6(17.1)	48(31.8)	0.085
Organ failure ^b^	13(37.1)	6(4.0)	<0.001^*^
Cardiovascular diseases ^c^	14(40.0)	43(28.5)	0.183
Pulmonary diseases ^d^	8(22.9)	14(9.3)	0.051
Digestive diseases ^e^	17(48.6)	36(23.8)	0.003^*^
Diabetes mellitus	5(14.3)	27(17.9)	0.612
Nephropathy ^f^	10(28.6)	20(13.2)	0.026^*^
Anemia	8(22.9)	29(19.2)	0.626
Rheumatism	3(8.6)	5(3.3)	0.358
Others ^g^	1(2.9)	7(4.6)	0.996
**Comorbidities ≥3**	24(68.6)	50(33.1)	<0.001^*^
**Indwelling catheter**	29(82.9)	112(74.2)	0.280
Serum indicators			
NLR	14.55 (6.50~22.97)	12.71 (6.78~23.22)	0.409
Percentage of lymphocytes (%)	6.30(4.00~12.90)	6.80(3.80~12.30)	0.680
CRP (mg/L)	125.17(98.92~161.79)	90.94(53.28~154.43)	0.009^*^
CRP≥98.28mg/L	27(77.1)	69(45.7)	0.001^*^
ALB (g/L)	28.87(25.70~32.36)	32.53(29.03~35.70)	0.001^*^
ALB ≤ 30.05g/L	22(62.9)	46(30.5)	<0.001^*^
LDH (U/L)	323.00(190.00~642.00)	208.30(162.70~298.90)	0.005^*^
LDH≥288.1U/L	21(60.0)	39(25.8)	<0.001^*^
BUN (mmol/L)	8.78(5.66~16.22)	5.58(4.01~8.04)	0.001^*^
BUN≥16.22mmol/L	11(31.4)	12(7.9)	<0.001^*^
Creatinine (μmol/L)	74.40(49.00~124.00)	67.00(49.60~84.30)	0.218
Creatinine≥127.3μmol/L	8(22.9)	12(7.9)	0.024^*^
Potassium ions (mmol/L)	3.90(3.39~4.40)	3.76(3.42~4.18)	0.440
Sodium ions (mmol/L)	136.40(132.10~139.20)	135.50 (132.20~138.70)	0.649
**Duration of antibiotic use**	12.0(4.0~21.0)	13.0(7.0~21.0)	0.285
**IEAT ^h^**	33(94.3)	140(92.7)	0.743
**IIAT ^i^**	4(11.4)	15(9.9)	0.792
**CRBSI ^j^**	6(17.1)	18(11.9)	0.406
**Drug-resistance**	26(74.3)	111(73.5)	0.925
**MDR ^k^**	14(40.0)	48(32.2)	0.381
**History of ICU admission**	12(34.3)	21(13.9)	0.004^*^
**Septic shock**	12(34.3)	7(4.6)	<0.001^*^
**Length of hospital stay**	25.0(18.0~37.0)	25(14.0~36.0)	0.996

a Genitourinary cancer included kidney cancer, bladder cancer, urothelial cancer, prostate cancer, ureteral cancer, cervical cancer, ovarian cancer, and endometrioid adenocarcinoma.

b Organ failure included respiratory failure, heart failure, liver failure, acute renal failure and multiple organ dysfunction syndrome.

c Cardiovascular diseases included hypertension, coronary heart disease, cardiac insufficiency, atrial fibrillation and pericardial effusion.

d Pulmonary diseases included chronic obstructive pulmonary disease, pulmonary hypertension, pleural effusion and interstitial lung disease.

e Digestive diseases included hepatic insufficiency, gastrointestinal bleeding, gastrointestinal obstruction, viral hepatitis, cirrhosis and ascites.

f Nephropathy included acute kidney injury and chronic kidney disease.

g others included hyperthyroidism, hypothyroidism, myelosuppression, syphilis and disseminated intravascular coagulation.

h IEAT, initial empirical antibiotic therapy.

i IIAT, inappropriate initial antibiotic therapy.

j CRBSI, catheter-associated bloodstream infection.

k MDR, multi-drug resistance.

^*^ P< 0.05.

### 3.2 Comparison between the mortality and survival cohorts

The follow-up time lasted 30 days after the occurrence of BSIs. The patients in the BSIs group were divided into the mortality group (n=35) and the survival (n=151). **Table 3** showed the clinical characteristics and laboratory data at the time of blood cultures of both groups. The percentage of liver cancer patients was significantly larger in the mortality group than that in the survival (34.3% vs. 16.6%, *P*=0.018). Compared with the survival group, there was markedly a larger portion of patients in the mortality group with stage III/IV (93.3% vs. 76.1%, P=0.035), lung/thorax infections (42.9% vs. 21.2%, *P*=0.008), peritoneal cavity (17.1% vs. 5.3%, *P*=0.042), digestive diseases (48.6% vs. 23.8%, *P*=0.003), ICU admission (34.3% vs. 13.9%, *P*=0.004) and septic shock (34.3% vs. 4.6%, *P*<0.001). As to laboratory findings, serum ALB (28.87g/L vs. 32.53g/L, *P*=0.001) level in mortality was significantly lower than that in survival, whereas serum C-reactive protein (CRP) (125.17mg/L vs. 90.94mg/L, *P*=0.009), LDH (323.00U/L vs. 208.30U/L, *P*=0.005) and BUN (8.78mmol/L vs. 5.58mmol/L, *P*=0.001) levels were relatively higher. Patients who received initial empirical antibiotic treatment (IEAT) accounted for 94.3% of patients in the mortality group and inappropriate initial antibiotic therapy (IIAT) accounted for 11.4%. The duration of antibiotic use in the mortality group was not noticeably different from that of the survival (12.0 days vs. 13.0 days, *P*=0.285). The total incidence of CRBSI was 12.9% (24/186). But no significant differences in the incidences of CRBSI (17.1% vs. 11.9%, *P*=0.406), IEAT (94.3% vs. 92.7%, *P*=0.743) and IIAT (11.4% vs. 9.9%, P=0.792) were discovered between the two groups.

### 3.3 Multivariate regression analysis of risk factors for BSIs incidence and death

**Figure 2:**
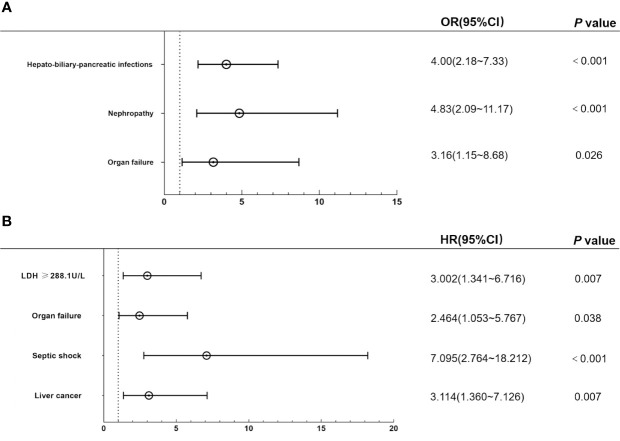
Multivariate regression analysis of the independent risk factors for incidence and mortality of BSIs. **(A)** Hepato-biliary-pancreatic infections, organ failure, and nephropathy were independent sensitive factors for BSIs in multivariate logistics regression analysis. **(B)** Liver cancer, septic shock, and organ failure were independent risk factors for death after BSIs in the multivariate Cox regression model. *BSIs*, bloodstream infections.

Multivariate logistic regression analysis revealed that organ failure (odds ratio [OR]: 3.16; 95% confidence interval [CI]: 1.15~8.68; *P*=0.026), nephropathy (OR: 4.83; 95% CI: 2.09~11.17; *P*<0.001), hepato-biliary-pancreatic infections (OR: 4.00; 95% CI: 2.18~7.33; *P*<0.001) were independent sensitive factors for patients with BSIs in solid tumours (**Figure 2A**). While liver cancer (hazard ratio [HR]: 3.114; 95% CI: 1.360~7.126; *P*=0.007), septic shock (HR: 7.095; 95% CI: 2.764~18.212; *P*<0.001), organ failure (HR: 2.464; 95%CI: 1.053~5.767; *P*=0.038) and LDH≥288.1U/L (HR: 3.002; 95% CI: 1.341~6.716; *P*=0.007) were independent risk factors for mortality after BSIs in patients with solid tumours according to multivariate Cox regression analysis (**Figure 2B**).

### 3.4 Construction of nomogram predicting risks of death after BSIs

**Figure 3:**
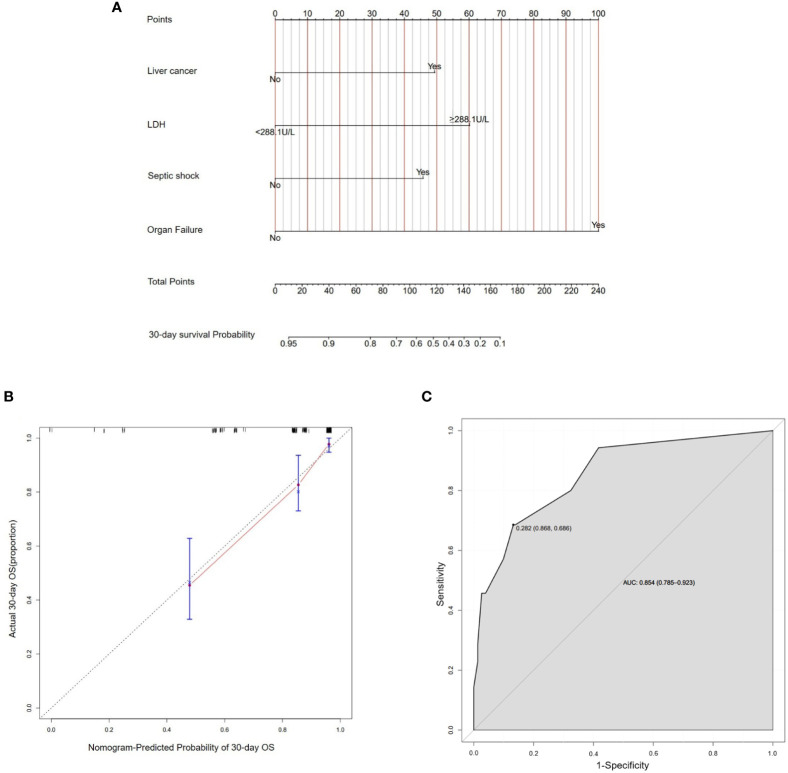
Establishment of nomogram about 30-day survival probability after BSIs. **(A)** A nomogram as a predictive model of BSIs in solid tumors. **(B)** In the calibration curve, the Y-axis represented the real-world probability of death after BSIs in solid tumors, and X-axis represents the predicted probability in this study. **(C)** The ROC curve represented the accuracy of the prediction model of BSIs (AUC = 0.854[0.785-0.923]). *BSIs*, bloodstream infections.

Based on these risk factors including liver cancer, LDH≥288.1U/L, organ failure and septic shock from multivariate Cox regression analysis, we established a nomogram that could predict the probability of 30-day survival of BSIs in solid tumors (**Figure 3A**). In the nomogram, we could find the score of each item through the vertical line in “Points” and calculate the total scores. Then the probability could be found by the total scores corresponding to the vertical line. The length of each variable was represented for its contribution degree to the outcome. In the calibration curve (**Figure 3B**), the slope =1 straight light indicated an assumption that the real world is in line with the predicted value. The red line deviated from the dotted line slightly. The ROC curve (**Figure 3C**) showed the AUC in the model was 0.854, which illustrated the prediction model has an excellent predictive ability.

## 4 Discussion

The clinical characteristics and risk factors of patients with BSIs in cancers were unclear (Al-Otaibi et al., 2016; Zhao et al., 2020). In our cohorts, the incidence of BSIs was 2.0/1,000 admissions (206/102,704), and the 30-day mortality rate was 18.8% (35/186). Multivariate logistic regression analysis showed that organ failure, nephropathy, and hepato-biliary-pancreatic infections were independent risk factors for BSIs in patients with solid tumors. Furthermore, we obtained that liver cancer, LDH≥288.1U/L, septic shock and organ failure were risk factors for death after BSIs in the multivariate Cox regression model. The four predictors were selected to construct a nomogram to predict the 30-day survival probability of BSIs. The results prompted clinical physicians to focus more on those patients with the above conditions during BSIs.

Similar to the study of Royo-Cebrecos et al. (Royo-Cebrecos et al., 2017a), hepato-biliary-pancreatic infections were independent risk factors for the occurrence of BSIs in solid tumors patients. The abdomen was one of the most common sources of BSIs because a majority of patients with hepato-biliary-pancreatic tumors carry biliary stents or drainage tubes (Royo-Cebrecos et al., 2017b; Vesteinsdottir et al., 2022). When the shifting stents or unsuccessful drainage causes biliary obstruction, the biliary microenvironment becomes a good culture medium for bacteria, such as *Enterococcus faecalis* (Wulkersdorfer et al., 2017). As a result, these patients were predisposed to have hepato-biliary-pancreatic infections. Renal failure and acute cholecystitis had been illustrated as the risk factors for BSIs in patients who have received hepato-biliary-pancreatic surgery (Yang et al., 2019). Furthermore, organ failure and nephropathy also were sensitive factors for BSIs. In clinical circumstances, we ought to be more mindful of indicators of liver and kidney function. If a patient with solid tumors accompanies hepato-biliary-pancreatic infections, it is crucial for clinician to watch out for hints of BSIs.

Concerning tumor types with BSIs, liver cancer was predominant. The incidence of liver cancer was increasing over the years (Cocker et al., 2019). Besides, we found liver cancer is a hazardous factor for BSIs. Since the liver is the organ with the largest amount of blood flow in the whole body, which greatly increases the chance of microorganisms residing in the biliary tract and then invading blood circulation. The physical condition of patients with liver cancer is poor. They are prone to be accompanied by hypoproteinemia, bile duct obstruction, jaundice, and even liver failure. All the above risk factors will cause an imbalance of the immune microenvironment, which is easy to induce BSIs. It suggests that if a patient with liver cancer develops symptoms of infection, it is essential to perform multiple blood cultures for confirmation and apply empirical antibiotic therapy timely.

In other types of solid tumors, a high level of LDH was observably associated with a worse prognosis (Chen et al., 2023; Deng et al., 2018; Blas et al., 2021). According to the report of Deng, et al. (Deng et al., 2018), the higher the baseline LDH level before treatment, the higher the overall mortality of patients with lung cancer. As an indicator of inflammation, LDH reflects the severity and increases the risk of infections. The value of the LDH to ALB ratio can be used as an independent prognostic factor for mortality in patients with lower respiratory tract infection (Lee et al., 2022). The incidence of organ failure in patients with acute pancreatitis with high levels of LDH was 4.38 times higher than that of patients with low levels. Except for liver cancer and high level of LDH, organ failure and septic shock also were risk factors for death of BSIs in solid tumors. In Vietnam, organ dysfunction is associated with a high case fatality rate of community-acquired BSIs (Dat et al., 2018). Sequential organ failure assessment score (> 5) was closely related to 28-day mortality of nosocomial BSIs (Jiang et al., 2019). Among ICU patients, the independent risk factor for 30-day death involved solid tumors, septic shock and renal failure (Zhao et al., 2020; Ababneh et al., 2022). The 30-day mortality with renal failure in ICU patients was 10.0%. The result was consistent with the reports of Royo-Cebrecos, Amanati, and Zhang Q, et al. (Royo-Cebrecos et al., 2017a; Zhang et al., 2019; Amanati et al., 2021). The above findings urge that we should pay more attention to the underlying diseases and primary infection sites of patients with solid tumors in clinical practice. Identifying predictors of mortality is essential to improve the clinical outcomes of patients with BSIs.

Few studies have comprehensively analyzed the epidemiology and risk factors of patients with BSIs in solid tumors. The investigation we performed aimed to enrich the theme and explore the clinical prognosis of that special population. What’s more, we established a predictive model to estimate the likelihood of 30-day survival of BSIs in solid tumors. Recently, plenty of studies formulated prediction models to explore the risk factors of disease progression and overall survival in cancer patients. In the risk score model developed by Yang, et, al (Yang et al., 2019), BSIs probability was described by different risk groups toughly through cumulative risk scores. In their report, the ROC with AUC was 0.72. Because of visualizing results, the nomogram is a popular and novel prediction model in recent years. But only a handful of nomogram models about BSIs in malignant tumors (Luo et al., 2022; Chen et al., 2018; Min et al., 2021; Tian et al., 2021), let alone solid tumors. As far as we know, there was only one study for BSIs in patients with solid tumors (Li et al., 2021). According to that research, the crude 30-day mortality of invasive candidiasis was 28.0%. The model was based on multivariable logistic regression analysis and predicted the 30-day death probability after invasive candidiasis. The ROC curve for the training cohort with AUC was 0.895. In our study, we established a nomogram to predict the 30-day survival of patients with BSIs in solid tumors. The specificity of ROC reached 0.854, which is quite excellent and indicates the potential for applying in practice. However, this predictive model needs to be verified and improved in future clinical studies.

In terms of drug resistance, we found the antibiotic resistance rates in GNB were 83.1% (101/124), while in GPB were 69.8% (37/53), which were incredibly high. Two retrospective studies(Freire et al., 2016; Royo-Cebrecos et al., 2022) reported that IIAT was associated with a high mortality rate in STs. Another study concluded that MDR increases the risk of death in patients with *Pseudomonas aeruginosa* BSI(Zhao et al., 2020). Although in our work, the difference between death and survival groups in IIAT (11.4% vs. 9.9%, *P*=0.792) and in drug-resistance (74.3% vs. 73.5%, *P*=0.925) was not significant, we detected that the total resistant rates were 71.9% (137/192) and proportions of drug-resistance and MDR in the death group were higher than the survival. It warns us to adopt strict measures to reduce the inappropriate use of antibiotics in the future and consider the epidemiological characteristics of local drug resistance patterns when initiating antimicrobial therapy.

Nevertheless, there are some limitations in our study. Firstly, it is a single-center retrospective study, the data obtained through historical cases from electronic medical record systems are possibly missing certain observation indexes, leading to failure in analyzing some indicators. Secondly, we involved 186 cases of BSIs, which was limited so that it can’t be refined the variables further. Last but not least, there were 206 cases of BSIs actually, of which 20 cases were removed by PSM. This may recede the statistical effectiveness. We look forward to multi-center prospective studies being planned to overcome those shortages in the future.

Conclusively, BSIs threaten the survival of patients with solid tumors. Special attention should be paid to patients who also have nephropathy, organ failure, hepato-biliary-pancreatic infections, liver cancer, or septic shock. By establishing a nomogram to effectively predict survival, we hope that the patients with BSIs in solid tumors could be better monitored and the clinical prognosis of BSIs can be improved ultimately.

## Data availability statement

The raw data supporting the conclusions of this article will be made available by the authors, without undue reservation.

## Author contributions

XL and JZ contributed to conception, design and validation of the study. LX collected the original data and wrote the first draft of the manuscript. YZ and MZ participated the data curation, investigation. PJ proofread the manuscript. LX and JF analyzed the data. All authors contributed to the article and approved the manuscript.
